# Tetrakis(2,2,6,6-tetramethyl-3,5-heptanedionate) Cerium for the Deposition of Hydrophobic Coatings

**DOI:** 10.1021/acs.langmuir.5c01723

**Published:** 2025-06-11

**Authors:** Jayna K. Patel, Iqra Ramzan, Cesar III De Leon Reyes, Ivan P. Parkin, Claire J. Carmalt

**Affiliations:** Materials Chemistry Centre, Department of Chemistry, 4919 University College London, London WC1H 0AJ, United Kingdom

## Abstract

Cerium oxide thin films represent an important approach to hydrophobic, self-cleaning solar modules, offering significant potential for photovoltaic applications. These coatings provide a durable, long-lasting alternative to conventional self-cleaning surface technologies, which often rely on polymer-based films prone to degradation over time. Here, we present the synthesis of tetrakis­(2,2,6,6-tetramethyl-3,5-heptanedionate) cerium ([Ce­(thd)_4_]) for the deposition of hydrophobic cerium oxide thin films by aerosol-assisted chemical vapor deposition (AACVD). The use of [Ce­(thd)_4_] as a precursor led to the deposition of cerium oxide coatings onto fluorine-doped tin oxide (FTO)-coated glass substrates. Water contact angles (WCA’s) of 91–101.1° were observed for films deposited at temperatures of 400–500 °C. Investigation into the influence of the substrate on the hydrophobicity of cerium oxide films demonstrated that FTO-coated glass offers a more favorable surface morphology compared to that of barrier glass. The deposited cerium oxide thin films were characterized by surface analysis techniques for the different deposition temperatures. X-ray photoelectron spectroscopy (XPS) analysis of the adhered thin films revealed a transition from a mixed-phase system of Ce^3+^ and Ce^4+^ to a composition dominated exclusively by Ce^4+^.

## Graphical abstract

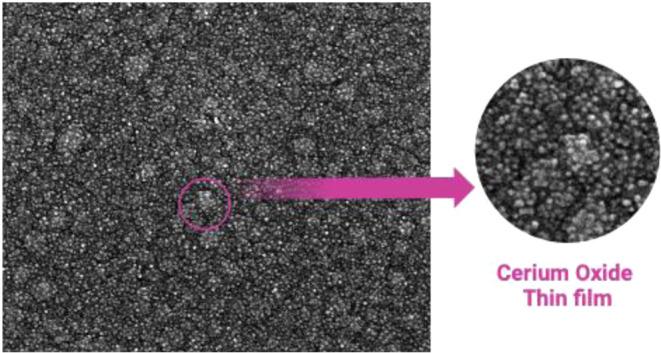

## 1 Introduction

Hydrophobic surfaces, characterized by high water contact angles of ≥90°, low surface energies, and low heats of immersion, play a crucial role in self-cleaning technologies.^1,2^ When water comes into contact with the surface, droplets form due to water repellency, reducing adhesion and enabling the removal of impurities from the surface. This phenomenon is predominantly associated with the “Lotus effect”, observed in nature, where water droplets rolling off lotus leaves remove dust and contaminants.^3–5^ While the pronounced rolling motion is characteristic of superhydrophobic surfaces, hydrophobic coatings still provide a self-cleaning mechanism by minimizing water adhesion and promoting droplet runoff. Inspired by these natural properties, hydrophobic surfaces offer a promising approach for enhancing the efficiency and durability of solar modules by reducing surface contamination.

Rare earth oxides have proven through various applications to be oxidant and ultraviolet (UV) resistant and have been recognized for their marked durability and hydrophobicity.^6–12^ Coatings of rare earth oxides are most commonly achieved by using atomic layer deposition (ALD) techniques.^13–17^ However, reported routes to lanthanide oxide coatings using ALD usually entail an extended deposition time.^18^ Likewise, residual contamination from elements such as carbon, hydrogen, and chlorine remains a large interference when depositing films using this technique, hindering the coating’s performance.

Aerosol-assisted chemical vapor deposition (AACVD) relies on the solubility of precursors for the formation of a precursor mist, which is deposited onto the desired surface.^19^ This technique enables control over the surface morphology and crystal structure of the resultant film by varying the deposition temperature and rate.^20,21^ The precursor is dissolved into a suitable solvent and vaporized by a nebulizer to create an aerosol mist. This mist is then carried by a preferred carrier gas into the reaction chamber composed typically of two parallel glass plates separated by approximately 8 mm. Here, the precursor particles are deposited, and the solvent is evaporated due to the heat of the chamber (dependent on the preferred deposition temperature).^21,22^ The substrate in AACVD can be either the top or bottom plate in the reaction chamber, subject to the type of precursor used. For the purposes of this investigation, the bottom glass plate is considered the substrate.

Polymer-based coatings are important for hydrophobic and superhydrophobic thin films and have proven to be successful in protecting surfaces.^23–28^ However, polymeric hydrophobic coatings often decompose because of weathering. Physiochemical deterioration from weathering can occur in the form of swelling, cross-linking, oxidation, water absorption, and more.^29,30^ As a result, polymer-based hydrophobic coatings have limited chemical resistance, restricting the use of these coatings for solar modules in ever-changing environments. This can also be said for the general durability and robustness of polymer films in comparison to rare earth oxides.^31^

Among rare earth oxides, studies on cerium oxide coatings provide contrasting reports on the hydrophobicity of cerium oxide.^32,33^ Cerium oxide can exist in the III and IV oxidation states, which can make its surface reactive, polar, and inherently hydrophilic.^34,35^ Interaction of cerium oxide surfaces with water can result in the formation of hydroxyl groups, enhancing the hydrophilicity of the material. However, it is evident that cerium oxide does exhibit contrastingly water-repellent/hydrophobic behavior because of particular synthesis methods.^36–40^ When functionalized with organic or nonpolar groups, cerium oxide can behave hydrophobically. Likewise, the removal or absence of hydroxyl groups on the surface will inherently reduce the affinity of cerium oxide to water. Methods such as chemical functionalization and surface roughness enhancement alter the structural integrity of the cerium oxide layer upon surface treatment to become hydrophobic and be applied to corrosion-resistant and water-repellent materials.

Evidently, various conditions can alter the hydrophobic properties of cerium oxide. Exposure to air notably increases the hydrophobicity of a cerium oxide layer, which is correlated with the adsorption of carbon on the surface. Likewise, the coating method used can influence the surface structure and its water-repellent abilities. Those that involve heat treatment of the surface can result in a hierarchical structure, significantly enhancing the hydrophobicity of cerium oxide. Moreover, cerium oxide has proven to serve as a hydrophobic coating in various applications, with varying deposition methods and surface treatments affecting the degree of hydrophobicity achieved.^7,31^ These include ceria coatings prepared by air-plasma spraying with water contact angles of 131°,^41^ nanocomposite systems incorporating cerium oxide nanoparticles to achieve superhydrophobic coatings,^42^ and various ALD routes.^43–45^ Literature reports are also evident on the deposition of hydrophobic cerium films onto silicon substrates via sputtering techniques, with water contact angles ranging from 90 to 109°.^16,46^ Overall, these effects demonstrate the complexity behind the hydrophobic nature of the cerium oxide.

While a limited number of literature sources report the growth of rare earth cerium oxide films by AACVD,^47–51^ to date, there are no reports on the deposition of such thin films via AACVD that exhibit intrinsic hydrophobicity. Hence, the growth of cerium oxide films is still a novel development among materials chemists. As detailed above, the synthetic method can affect the surface chemistry of resulting films, and contrasting reports on the hydrophobicity of cerium oxide indicate that a detailed study on AACVD of CeO_2_ films is required in order to gain further insight into the hydrophobic nature and potential antisoiling properties of the films. This study investigates the synthesis of cerium oxide thin films via AACVD using a precursor complexed with an ancillary donor ligand.

## 2 Experimental Section

### 2.1 Materials and General Procedures

All chemicals were purchased from Merck (Sigma-Aldrich) and used without further purification. Preparations for the synthesis of the precursor used for deposition were performed under ambient conditions. The resulting product was stored in a vial at room temperature and sealed with a parafilm. FTO and barrier glass substrates (30 cm × 30 cm) were supplied by NSG Pilkington and cut to the appropriate size for AACVD (15 cm × 5 cm) for thin film deposition. The nitrogen cylinder was supplied by BOC Co. Ltd.

### 2.2 Precursor Synthesis

Tetrakis­(2,2,6,6-tetramethyl-3,5-heptanedionate) cerium [Ce­(thd)_4_] was prepared using a known synthesis reported by Becht et al., with adaptations.^52–54^ A solution of tetrakis­(2,2,6,6-tetramethyl-3,5-heptanedionate) (H­(thd), 1.46, 7.9 mmol) was prepared in 4 M NaOH (2.9 mL) and 96% ethanol (50 mL). A second ethanolic solution (50 mL) of cerium ammonium nitrate (CAN, 1.50 g, 2.7 mmol) was prepared and stirred for 10 min at 30 °C. The CAN solution was then added dropwise to the ligand solution and stirred for 20 min at 50 °C, yielding a dark brown solution. The product was collected by centrifugation (at 4500 rpm for 7 min) and dried in an open vessel at room temperature. The final product was attained as a brown powder with 80% yield. ^1^H NMR analysis, consistent with the literature, confirmed the synthesis of the desired product. Thermogravimetric analysis (TGA) of the cerium precursor (Figure S1) under nitrogen revealed an onset of decomposition at approximately 175 °C, with around 60% weight loss observed by 440 °C. These results indicated that complete volatilization and decomposition of the precursor occurs well below 500 °C. Based on this, a deposition temperature range of 450–500 °C was selected to ensure efficient transport and thermal decomposition of the precursor during the AACVD process while avoiding premature degradation or incomplete reaction.

H NMR δ/ppm (CDCl_3_, 500 MHz): 1.20 (s, 18H, ^t^Bu), 5.53 (s, 1H, C­(=O)­CHO), 7.26 (CDCl_3_ peak).

### 2.3 AACVD

Each deposition was carried out using the same experimental setup except for varying deposition temperatures and type of glass substrate where mentioned. Depositions were carried out using a cold wall CVD horizontal bed reactor reported previously.^55^ The CVD reactor was assembled in a bottom-up heating configuration, whereby a carbon block was situated below the glass substrate parallel to the supporting glass plate 8 mm above. This system was enclosed by a quartz glass tube. The cerium precursor solution was prepared by dissolving 200 mg of the synthesized [Ce­(thd)_4_] precursor into 30 mL of toluene. An AACVD glass bubbler containing the precursor solution and connected to the N_2_ inert gas line was positioned above an ultrasonic humidifier and used to generate the aerosol mist carried to the reactor for deposition. Prior to the deposition and generation of the aerosol mist, the reactor and glass plates were heated to the appropriate deposition temperature under a reduced flow rate of dinitrogen at 0.8 L/min. During deposition, this flow was increased to 1.0 L/min, carrying the aerosol mist through the baffle to the reaction chamber for deposition on the glass substrate. Post deposition, the dinitrogen flow rate was lowered again to 0.8 L/min while the apparatus was left to cool. The final treated glass samples were handled and stored in air.

Notably, postdeposition annealing in air did not improve the films; instead, it led to material loss at lower temperatures and structural degradation at higher temperatures. As this treatment also resulted in a significant decrease in the water contact angle, it was not pursued further.

### 2.4 Physical Characterization

Analysis of the thin films was carried out as synthesized. X-ray photoelectron spectroscopy data was acquired using a thermoscientific K-Alpha photoelectron spectrometer with the use of a monochromatic source of Al kα radiation at 1486.6 eV. Avantage XPS software was used for determining the binding energies of sample measurements with a charge correction calculated against carbon charge 284.6 eV. Grazing incidence X-ray diffraction (GIXRD) spectra of the deposited thin films were obtained using a Pananalytical GIA X-ray diffractometer with the following scan parameters: 2θ = 4–80°, 0.05 step, 0.5 s, ω = 1°. Scanning electron microscopy (SEM) images were captured by using a SEOL field emission SEM instrument. Additionally, prior to SEM analysis, the deposited films were cut to the appropriate size and vacuum sputtered with a thin layer of gold to enhance the surface electrical conductivity of the sample for analysis. Using a Shimadzu UV-2600 Spectrometer, ultraviolet-visible (UV–vis) transmittance, reflectance, and absorbance measurements were recorded within the range of 300–1000 nm. Finally, static water contact angle measurements were conducted using a Krüss DSAE Droplet Shape Analyzer by the sessile drop method.

## 3 Results and Discussion

AACVD of cerium oxide thin films onto glass substrates was investigated via the deposition of the synthesized precursor, [Ce­(thd)_4_]. The deposition was conducted in toluene. Thermogravimetric analysis of [Ce­(thd)_4_] indicated 50% mass loss by 270 °C. Thus, AACVD was conducted at substrate temperatures of 400–500 °C at intervals of 50 °C. Depositions carried out between 400 and 500 °C led to the growth of cerium oxide coatings on the surface of FTO-coated and silica-coated (barrier) glass substrates, with those deposited on FTO glass showing intrinsic hydrophobicity. The formation of cerium oxide results from the decomposition of the oxygen-containing tetravalent precursor used for deposition ([Ce­(thd)_4_]). It was observed that increasing the deposition temperature improved the hydrophobic nature of the film. The films deposited on fluorine-doped tin oxide (FTO)-coated glass substrates from [Ce­(thd)_4_] at 400, 450, and 500 °C will hereafter be referred to as samples **A400**, **A450**, and **A500**, respectively. Those carried out on barrier glass at the same corresponding temperatures will be referred to as **B400**, **B450**, and **B500**.

### 3.1 X-Ray Diffraction (XRD) Analysis

The XRD patterns of the FTO-coated films **A400**, **A450**, and **A500** are shown in Figure 1, in comparison to the standard XRD of pure Ce_2_O_3_ and CeO_2_, respectively.^56,57^ Among the deposited hydrophobic films (**A400**, **A450**, and **A500**), crystalline peaks primarily correlating to CeO_2_ were observed. The following diffraction peaks matched with the cubic structure of pure CeO_2_: (111), (200), (220), (311), and (420), as shown in Figure 1.^56^ At the lowest deposition temperature (**A400**), these peaks were largely shadowed by the highly crystalline nature of the FTO substrate itself. Nonetheless, with increasing deposition temperature and thicker films, there were less noticeable FTO peaks. With a 50 °C increase in deposition temperature from sample **A400** to **A450**, the correlating XRD pattern was more amorphous. Interestingly, when the temperature was then increased by another 50 °C (sample **A500**), there was a shift to a more crystalline XRD pattern again. This trend was also observed upon XRD analysis of the barrier glass-coated samples (**B400**, **B450**, and **B500**) upon increasing deposition temperature, as shown in Figure 2. Regardless, Figure 2 represents the successful synthesis of cerium oxide coatings and the retention of the lanthanide coating in the (IV) oxidation state while altering the substrate adopted for deposition.

**Figure 1:**
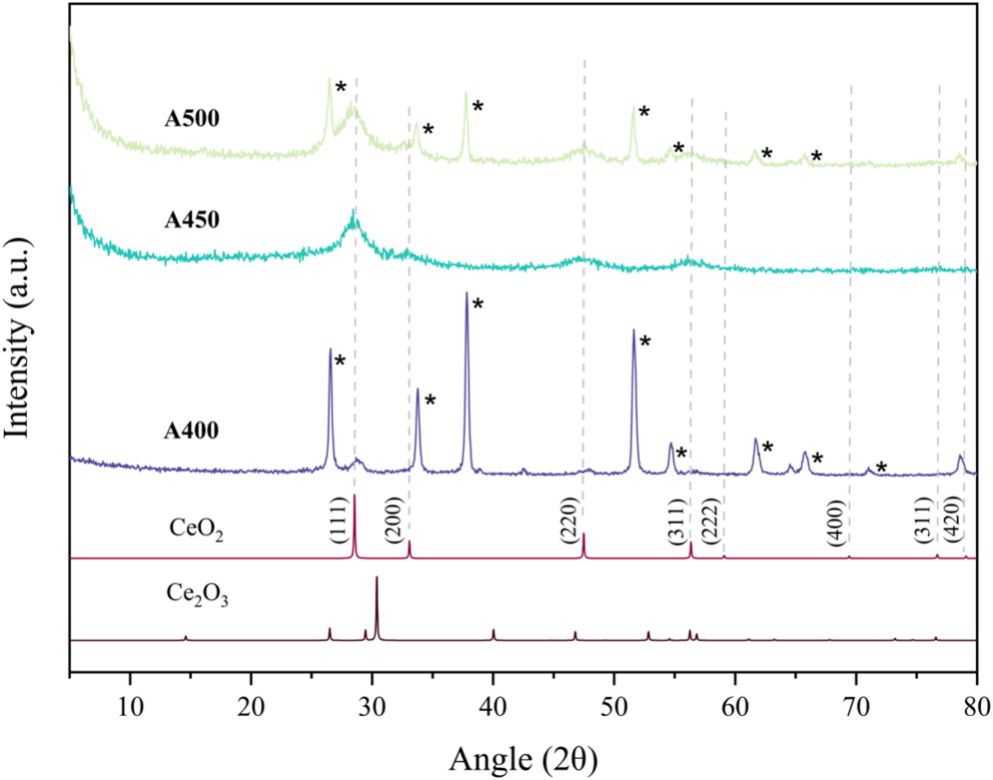
XRD patterns of deposited cerium oxide films (**A400, A450**, **and A500**) by AACVD of [Ce­(thd)_4_] onto FTO glass, recorded using GIXRD, against the XRD pattern of pure cerium­(III) and cerium­(IV) oxide. Reflections corresponding to the underlying FTO substrate are marked with an asterisk (*).

**Figure 2:**
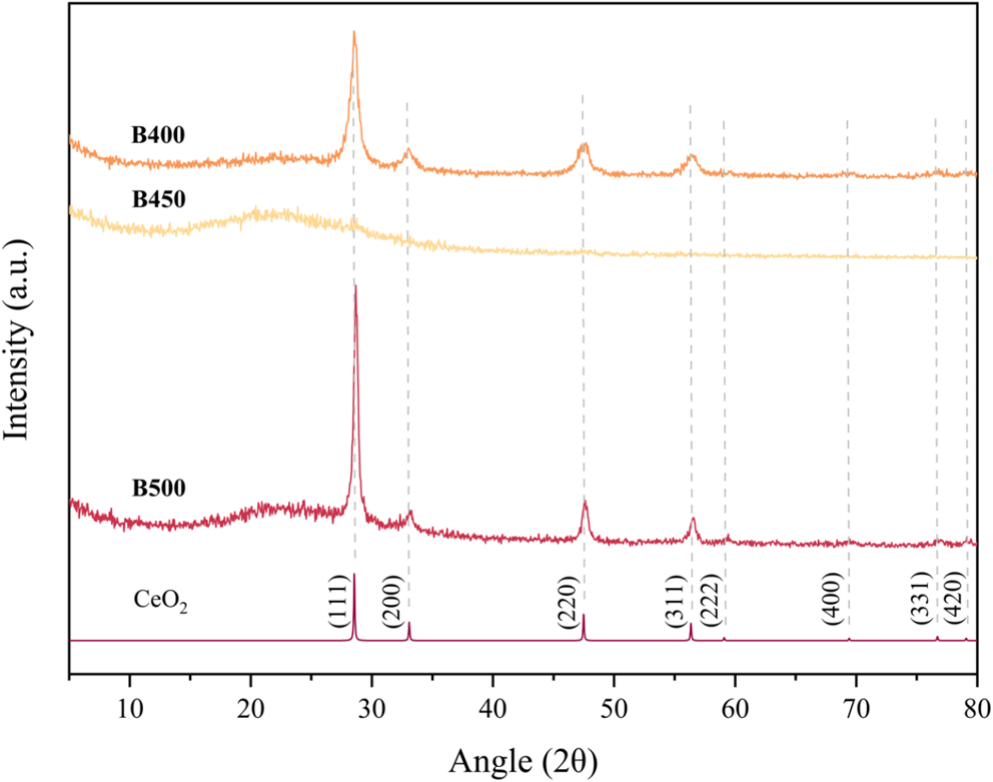
XRD patterns of deposited cerium oxide films (**B400, B450, and B500**) by AACVD of [Ce­(thd)_4_] onto FTO glass, recorded using GIXRD, against the XRD pattern of pure cerium­(IV) oxide.

In its solid form, cerium can exist in two main crystalline phases: Ce_2_O_3_ and CeO_2_.^58^ Evidently, the difference in patterns arises from their difference in oxidation states (Ce­(III) and Ce­(IV)).^59,60^ The data collected from this investigation provides evidence of the formation of CeO_2_ from the deposition of [Ce­(thd)_4_]. However, incomplete oxidation of the decomposed precursor can be identified from the presence of low-intensity peaks correlated to Ce_2_O_3_ from the XRD analysis of sample **A400**.

Figures 1 and 2 show the tendency of cerium­(IV) oxide films to grow in the (111) orientation. This is not uncommon for materials that adopt a cubic structure such as CeO_2_.^61–63^ Since the (111) orientation exhibits the lowest surface energy, it is more stable and thus energetically favorable for promoting growth in that direction. Likewise, this growth orientation can often be promoted by specific substrates or conditions. These include, but are not limited to, lattice matching, strain, atomic packing density, and other factors with implications on nucleation and growth dynamics. It is apparent that CeO_2_ coatings that are (111) highly oriented are likely to have different electrical or catalytic properties than those of a randomly oriented film. This study demonstrates the growth of CeO_2_ thin films from crystallites with a (111) preferred orientation, as seen in Figure 1.

X-ray diffraction analysis of the same experiments conducted on barrier glass substrates (samples **B400**, **B450**, and **B500)** shows the nature of the deposited cerium oxide film without interference from crystalline substrate peaks. This is a result of the amorphous diffraction pattern associated with barrier glass.^64,65^ Figure 2 shows the nanocrystalline nature of the deposited cerium oxide films. As previously mentioned, it is evident in the case of each substrate that deposition of cerium oxide via AACVD at 450 °C (**A450** and **B450**) delivers a more amorphous diffraction pattern compared with those at higher and lower temperatures. Possible reasoning for this could be that at 450 °C, the deposition of cerium oxide is at a transitional phase, where kinetic and thermodynamic factors relating to crystal growth are not fully aligned. Thus hindering the development of a fully crystalline film. Additionally, the broadness of these amorphous peaks could indicate smaller crystallite sizes or higher defect densities obtained at 450 °C as a result of kinetic competition.

Research conducted by Wu et al. details the XRD patterns of cerium nanoparticle coatings.^66^ Here, the diffraction patterns of bulk CeO_2_ and Ce_2_O_3_ nanoparticles display an array of broadened diffraction peaks due to the small crystallite size. Similarly, broadened peaks characteristic of those exhibited by samples **B400**, **B450**, and **B500** could imply the extent of how small the cerium particles are along the surface of the slightly more amorphous samples. Overall, XRD analysis confirms the successful deposition of cerium­(IV) oxide via AACVD of tetrakis­(2,2,6,6-tetramethyl-3,5-heptanedionate) cerium on FTO and barrier glass substrates.

### 3.2 XPS Analysis

XPS was used to analyze the oxidation states of cerium present across the depositions carried out on FTO, as well as the chemical environments of oxygen and carbon species apparent. As previously mentioned, cerium exists in two main oxidation states: Ce^3+^(cerous) and Ce^4+^(ceric).^67^ The Ce 3d spectra are known to be complex and detailed due to the potential for multiple electron transitions, resulting in spin–orbit doublets. Thus, very few reports are available that fully resolve the XPS of ceria.^7,68–70^

XPS surface analysis of the deposited films (**A400**, **A450**, and **A500**) on FTO revealed a mixture of elemental oxidation states (Figures 3, 4, and 5). Due to the spin–orbit doublets associated with cerium oxide, the photoelectron spectrum for Ce^3+^ 3d depicts two final states. The Ce 3d spectrum for pure Ce_2_O_3_ is composed of six visible components despite there being only one chemical state as a result of further multiplet splitting. Each of these is usually paired and separated by (ΔCe_2_O_3_ = 18.0 Ev). In order to distinguish the Ce­(III) from Ce­(IV) analysis of the multiplet, splitting is required as Ce­(IV) typically has an additional peak at 917 eV, which is absent in the spectrum for Ce­(III). High-resolution XPS analysis of the Ce 3d spectra revealed that the **A450** sample contained a significant fraction of Ce­(III) (39.8%) alongside Ce­(IV) (60.2%), indicating the presence of oxygen vacancies or surface hydroxyl groups (Figure 5). In contrast, the **A500** sample (as presented in Figure 5) showed an almost fully oxidized surface with Ce­(IV) dominating the spectrum (∼100%). Further reduction of the residual Ce­(III) at 450 °C upon increasing the deposition temperature aligns with the higher WCA observed across sample **A500**, suggesting enhanced surface hydrophobicity.

**Figure 3:**
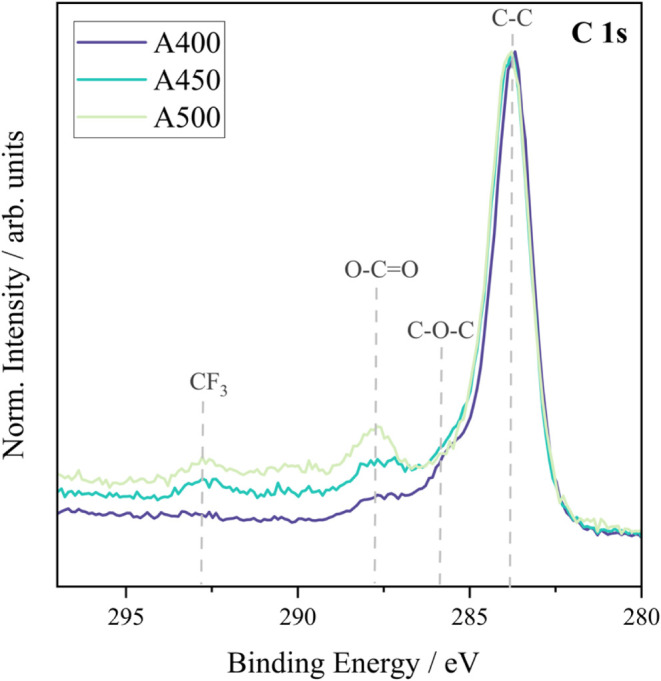
XPS spectra attained for the C 1s of samples **A400, A450, and A500** (cerium oxide films on FTO) measured using a monochromatic source of radiation.

**Figure 4:**
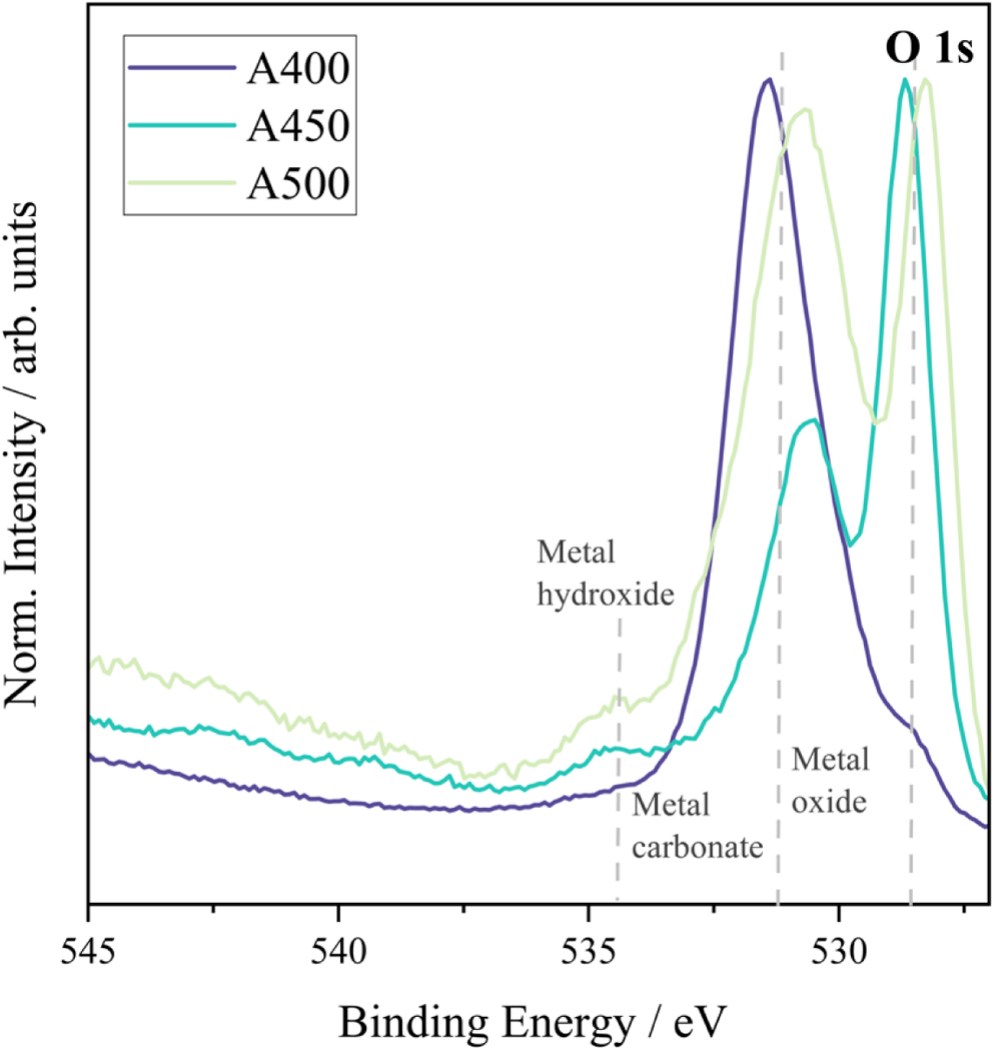
XPS spectra attained for the O 1s of samples **A400, A450, and A500** (cerium oxide films on FTO) measured using a monochromatic source of radiation.

**Figure 5:**
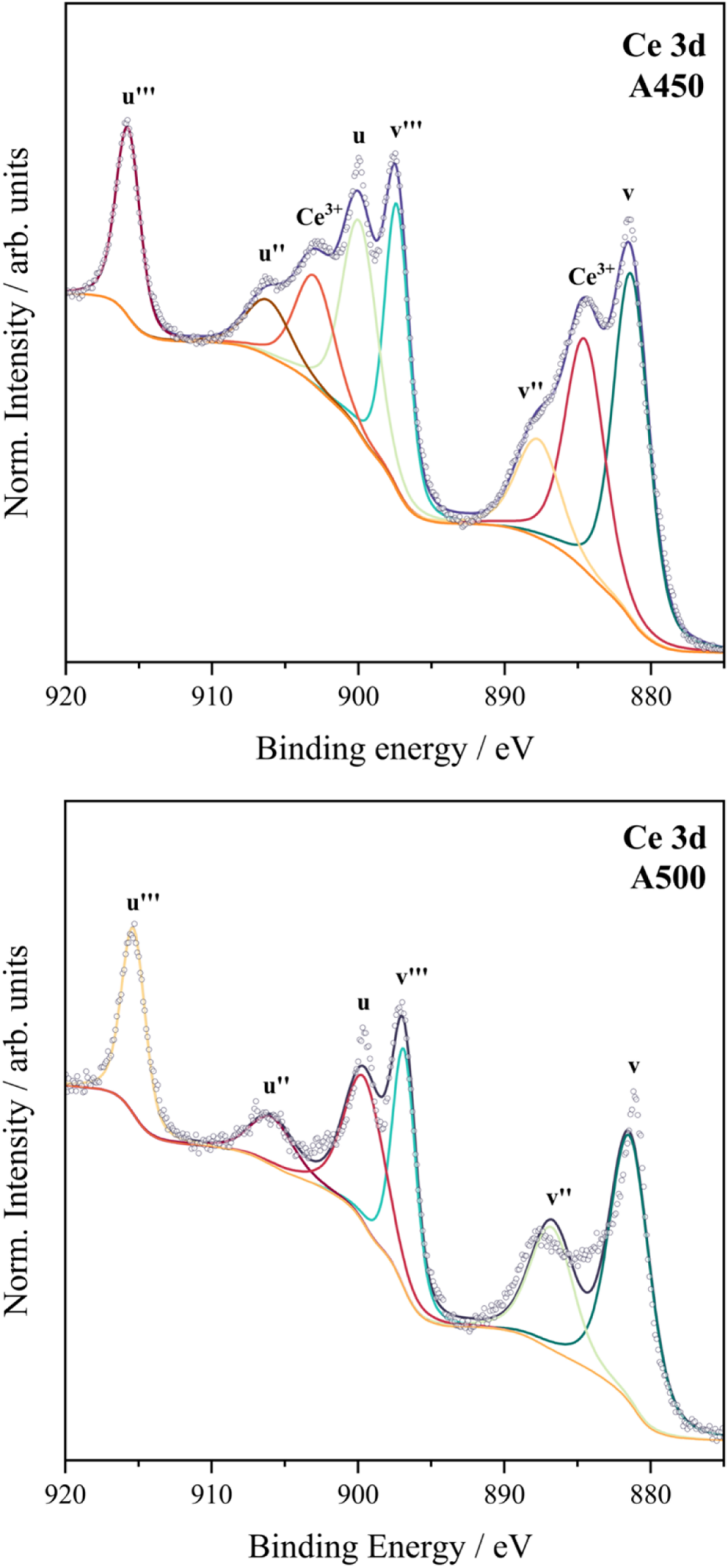
XPS spectra attained for Ce 3d of samples **A450** and **A500** (cerium oxide films on FTO) measured using a monochromatic source of radiation.

These findings align with the O 1s spectrum for **A500** (Figure 4), which shows a higher intensity of metal carbonate species compared to **A450**, indicating that complete oxidation of Ce­(III) to Ce­(IV) occurs at 500 °C. The C 1s spectra (Figure 3) further confirm the presence of C–C, O–C=O, and C–O–C species arising from residual ligands, metal carbonates, and possibly adsorbed CO_2_, as discussed in Section 3.4. Together, the XPS data indicate a transition from a mixed Ce­(III)/Ce­(IV) phase in **A450** to a predominantly Ce­(IV) phase in **A500**. This change in surface chemistry strongly affects wettability: the higher Ce^3+^ content and associated surface defects in **A450** enhance hydrophilicity, while the Ce^4+^-rich surface in **A500** leads to increased hydrophobicity. These trends support the role of oxygen vacancies and Ce^3+^ species in promoting surface polarity and water adsorption, which diminish with higher deposition temperatures.

### 3.3 SEM Surface Morphology

The surface morphology of the films was studied using scanning electron microscopy at magnifications ranging from ×10,000 to ×33,000. The images in Figure 6 correspond to the surface morphologies present across the coatings for both the FTO and barrier glass-coated samples. Notably, a distinct difference can be seen across the treated surfaces when cerium oxide is deposited at increasing temperatures. Figure 6a shows the composition of the coating which delivered the lowest hydrophobicity (**A400**). Here, sparse clusters of cerium oxide nanoparticles are seen, with some larger clusters around 0.5 μm adding texture to the surface.

**Figure 6:**
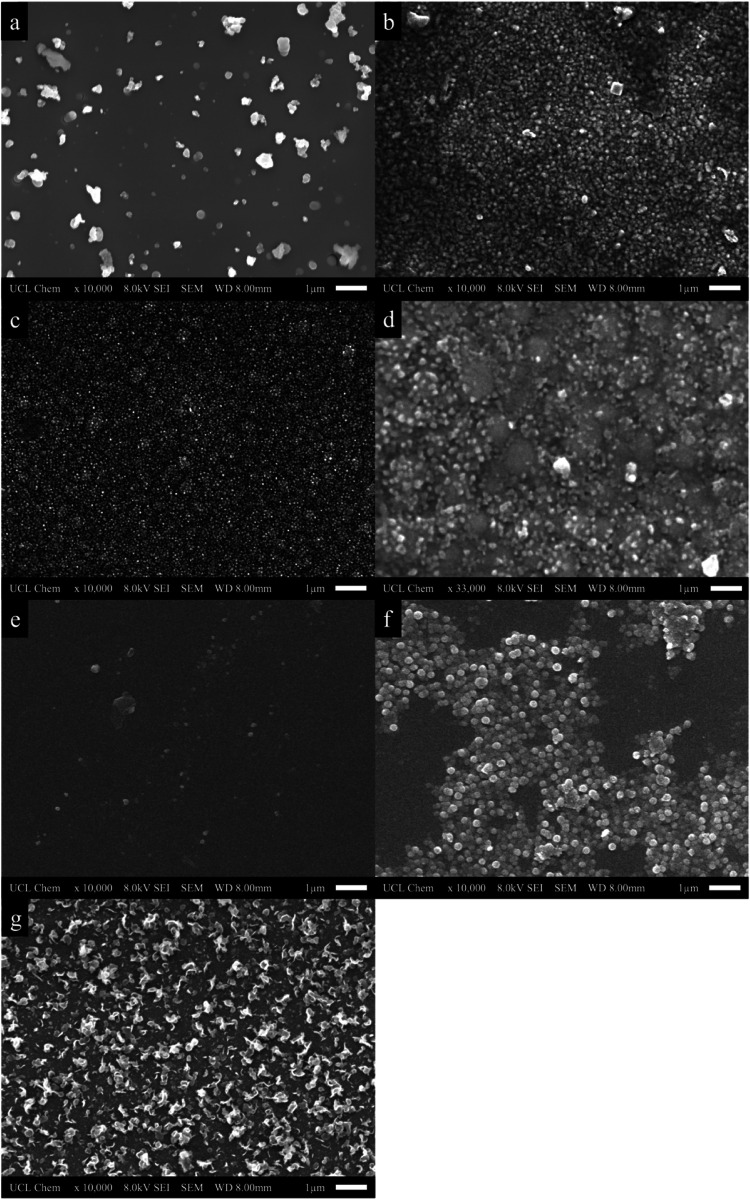
Surface morphology of (a) **A400**, (b) **A450**, (c) **A500**, and (d) **A500** at greater magnification, and (e) **B400**, (f) **B450**, and (g) **B500** cerium oxide films captured by SEM technology.

Literature reports that high substrate roughness provides more nucleation sites, which are essential for the growth of the deposited film.^3,11^ Thus, the textured FTO glass provides a perfect environment for the development of a hydrophobic surface. A significant difference in surface morphology was observed between samples **A400** and **A500**. The growth of ceria agglomerates was observed on the surface along with their aggregation until they are compact at higher temperatures, such as **A450** (Figure 6b). Figure 6c depicts the granular structure observed across sample **A500**, where closely packed grains are visible across the substrate with varying degrees of surface roughness. This provides a homogeneous and rough surface texture with properties that mirror those necessary for a surface to display intrinsic hydrophobicity.^1,2^ We can estimate the size of the particles to be between 10 and 50 nm. The formation of the closely packed grains captured in this study is similar to those reported by Chen and Wang,^71^ where a sputtering technique was used for the deposition of cerium oxide to give a compact granular surface morphology like that achieved on **A450**.

The SEM images reveal that while **A400** is less homogeneous and compact than **A450** and **A500**, ceria clusters are still identifiable. Higher deposition temperatures promote particle sintering, leading to denser films and increased surface texturing. This indicates that the ceria film morphology on FTO can be fine-tuned by adjusting the deposition temperature during AACVD of [Ce­(thd)_4_]. The spherical particle shape suggests isotropic growth, with **A500** exhibiting the most uniform structure, aligning with the increased crystallinity observed in Figure 6a–c. Päiväsaari et al. describe the deposition of erbium oxide [Er­(CpMe)_3_] thin films using ALD and the morphology of the deposited films using atomic force microscopy (AFM).^72^ Comparatively, their lanthanide oxide films appeared to have a conformal smooth surface morphology and an increase in surface roughness with an increasing deposition temperature. They identified the increase in surface roughness to correlate with an increase in the crystallinity of the films, consistent with the findings in this study.

Upon comparison of the morphologies observed for the samples deposited on FTO and those deposited on barrier glass, it is clear that the substrate influence plays a key role in the overall crystallinity and morphology of the film. The transition of morphology from samples **B400** to **B450**, as displayed in Figure 6e,6f, further provides evidence of higher deposition temperatures, creating a more densely packed coating. Fine-tuning of the surface between the two samples is also apparent as ceria nanoparticles that were irregular in shape and size became more spherical and uniform as the deposition temperature was increased from **A400** to **A500**. Contrastingly, the surface morphology correlating to sample **B500**, shown in Figure 6g, shows an irregular petal-like structure across the surface of the plate, whereas the deposition carried out on FTO led to a highly dense, compact granular structure (**A500**). This difference is most likely due to the ability of ceria particles to grow across the blank substrate laterally in a two-dimensional direction in sample **B500**. On the other hand, the grooves apparent from the natural crystalline surface of FTO forces ceria grains to grow isotopically to form a densely packed surface enhancing its ability to have intrinsic hydrophobicity.

### 3.4 Water Contact Angle Measurements

From the analysis described so far, it is evident that AACVD of [Ce­(thd)_4_] at increasing temperatures between 400 and 500 °C results in complete oxidation of ceria on the surface of FTO, providing a highly crystalline and compact granular surface structure. This aligns with the trend of increasing water contact angles (WCAs) observed in Table 1. Here, the increasing hydrophobicity of samples **A400**, **A450**, and **A500** was observed, with **A500** showing the highest hydrophobic water contact angles of up to 101.1° ± 0.21 on FTO at 500 °C. Depositions carried out using the same fixed parameters at temperatures above 550 °C led to an exponential decline in hydrophobicity. Typically, bare FTO and barrier glass plates are hydrophilic with WCAs of 41.1° ± 1.1 and 27.9° ± 1.8, respectively. Thus, the results from this investigation demonstrate an improvement in the water contact angle by depositing cerium oxide of approximately 60° for FTO and 40° for barrier glass. Both of these show an increase of water contact angle when depositing ceria between 400 and 500 °C. Advancing and receding contact angle measurements revealed increasing hydrophobicity with deposition temperature, with the highest advancing angle (121°) observed for A500 (as shown in Table S1). However, contact angle hysteresis (CAH) also increased significantly across the series, from 37.0° (**A400**) to 75.6° (**A500**). This suggests that while higher deposition temperatures promote hydrophobicity, the films also exhibit stronger contact line pinning and reduced water mobility, likely due to increased surface roughness or heterogeneity.

**Table 1:**
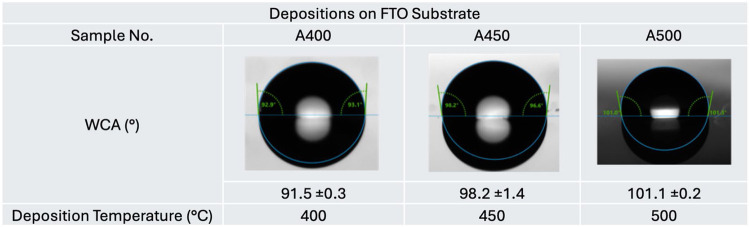
WCA Measurements of Hydrophobic Cerium Oxide Coatings Deposited onto FTO Glass (**A400**–**A500**)

The deposited films appeared uniform and continuous with a dark brown tint that became more pronounced with increasing deposition temperature. This effect was most noticeable in the films deposited on FTO substrates (**A400–A500**), while those on barrier glass (**B400–B500**) exhibited a much lighter tint. The intensification of the brown coloration is likely associated with increased incorporation of carbon-based species such as metal carbonates or residual organics, as observed in the C 1s and O 1s XPS spectra. These results suggest that both the substrate and deposition temperature influence the extent of surface contamination, which in turn affects the films’ optical appearance and wetting behavior.

Interestingly, the values presented in Table 2 indicate a decrease in hydrophobicity attained when depositing the same tetravalent cerium precursor onto barrier glass substrates, compared to that on FTO-coated glass (Table 1). This is likely due to the substrate FTO having a higher initial surface roughness compared to barrier glass. Notably, a high surface roughness allows higher surface energy and increased nucleation sites,^73,74^ allowing an optimal environment for the growth of a hydrophobic surface.

**Table 2:** WCA Measurements of Hydrophobic Cerium Oxide Coatings Deposited onto Barrier Glass (**B400**-**B500**) Taken Using Kruss DSAE

depositions on a barrier glass substrate			
sample no.	B400	B450	B500
WCA (°)	60.4 ± 2.4	63.9 ± 2.7	66.0 ± 2.1
deposition temperature (°C)	400	450	500

Literature on the deposition of lanthanide oxides reports improved water contact angles upon exposure of the treated surfaces to the atmosphere.^11,12^ This is also true for these hydrophobic films deposited on FTO (**A400**, **A450**, and **A500**), where once the samples had been exposed to the atmosphere for 30 days, enhanced water contact angles were observed, as shown in Figure S2. Lundy et al. conclude that the hydrophobic characteristics of rare earth oxides are governed by the adsorption of organic species that alter the surface energy and wettability of such materials.^33^ Evidently, adsorption of long-chain alkanes influences the surface chemistry of rare earth thin films by providing improved stability upon absorption and thus increasing the hydrophobicity of the surface.^12^ Thus, the improved water contact angle established in this study is the result of hydrocarbon adsorption from the atmosphere, enabling saturation of the ceria thin films.^11,31^ Overall, the research conducted to develop these thin films demonstrates that their hydrophobicity increases after 30 days of exposure to ambient air, making them even more effective and suitable for applications in photovoltaic systems (PVs). Additionally, deterioration of transmittance of the films was not exhibited on standing in the air during this period. This enhanced hydrophobicity ensures better durability and performance, which are critical for optimal energy efficiency.

### 3.5 Optical Measurements

UV–visible measurements enabled optical analysis of the deposited films (A400–A500) compared with the bare substrate (FTO). Transmittance and absorbance measurements of the hydrophobic samples were recorded between 260 and 1400 nm, as shown in Figures 7 and S5, and barrier glass depositions (Figures 8 and S6), respectively, where the visible region (380 and 700 nm) to the near-infrared region is highlighted in each plot. The reflectance of the samples was calculated under the assumption that all incident light is either transmitted, absorbed, or reflected. Therefore, the sum of absorbance, transmittance, and reflectance is equal to 100%. The reflectance of all of the samples can be seen in Figures S3 and S4, respectively. Typically, the FTO exhibits an optimal transmittance of approximately 80% in the visible region. Likewise, doping tin oxide with fluorine to give FTO induces a decrease in optical transmittance within the UV–vis range and consequently an increase in transmittance above 660 nm.^75,76^

**Figure 7:**
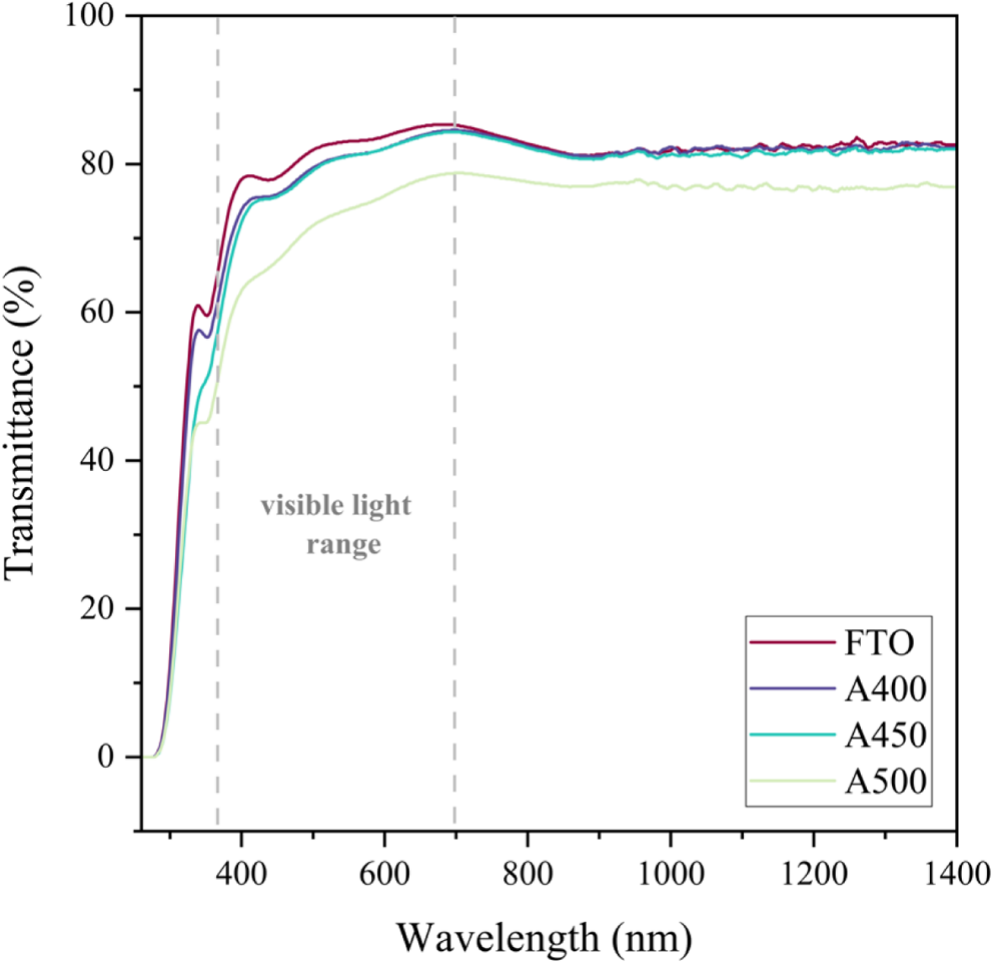
Optical transmittance measurements for samples **A400**, **A450, and A500** against that of bare FTO, with the visible light range (380–700 nm) highlighted.

**Figure 8:**
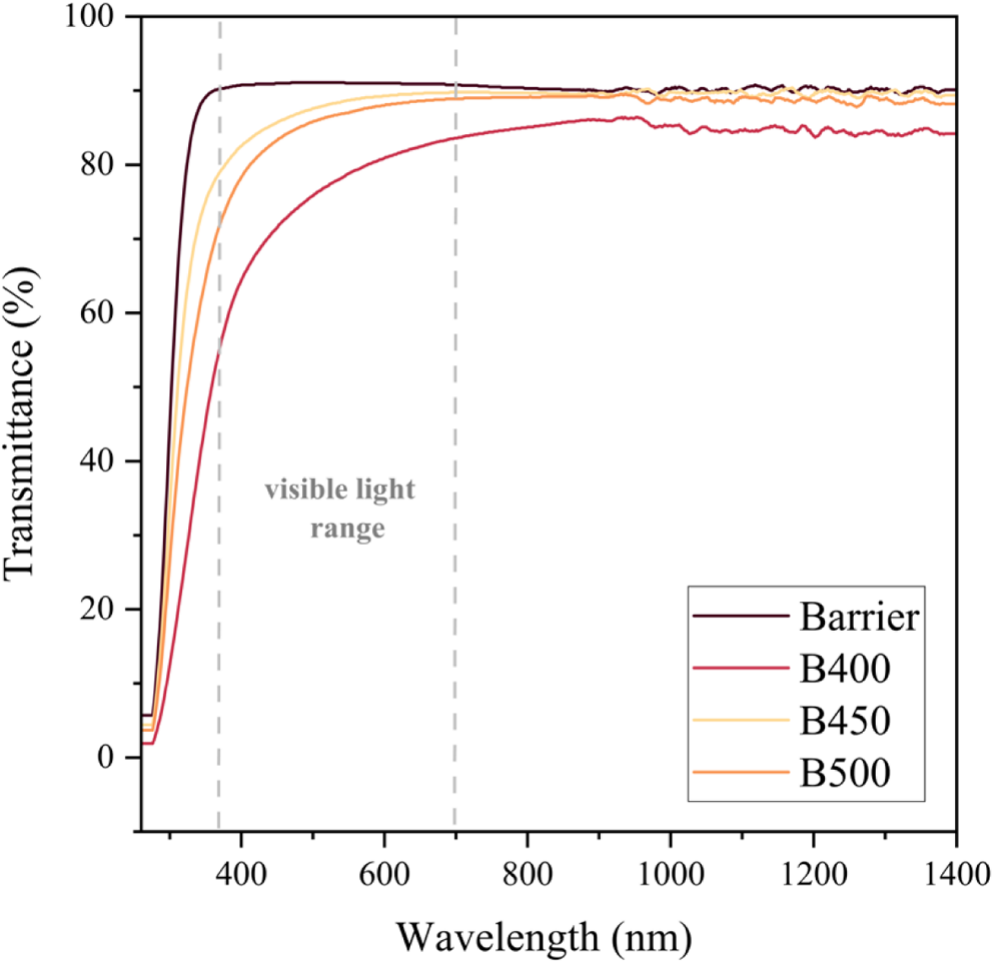
Optical transmittance measurements for samples **B400, B450, and B500** against that of bare barrier glass, with the visible light range (380–700 nm) highlighted.

From this study, it is evident that the transmittance decreases upon treatment of the FTO surface with cerium oxide. It is also apparent that with increasing deposition temperature and hydrophobicity of the films, the transmittance of the sample decreased due to film thickness across the treated glass plates, as displayed by the brown tint across the treated substrates. Sample **A500** had the lowest optical transmittance and highest reflectance and corresponding water contact angle due to increased film thickness at the highest deposition temperature.

A comparison of the optical properties of all of the samples deposited on FTO-coated glass and barrier glass allows several conclusions to be drawn. Evidently, FTO glass has a higher standard absorbance than barrier glass (as shown in Figure S6) because of the presence of an additional doped layer. The doped layer, due to FTO, contains a high density of free electrons, enabling the conductivity of the glass and enhancing its optical and electrical properties. Interaction of this doped layer with light allows some wavelengths of light to be absorbed by free-carrier absorption and scattering effects. On the other hand, the barrier glass used for depositions in this investigation is a plain substrate with only a thin SiO_2_ layer (c 50 nm) coating. With no additional doped layer to absorb light, the extent to which samples grown on barrier glass (**B400**, **B450**, and **B500**) can absorb light in comparison to samples on FTO (**A400**, **A450**, and **A500**) is hindered. Furthermore, the described textured nature and increased thickness of FTO glass provide an ideal surface morphology for trapping light and enhanced absorbance at the surface of samples deposited on FTO (Figure S5) over samples on barrier glass (Figure S6).

Comparatively, the transmittance of samples grown on barrier glass was inherently higher than those grown on FTO substrates due to the high transmissivity of barrier glass. The clear and uniform nature of the barrier glass enables light to pass through with minimal absorption and scattering. Thus, the deposition of a tinted lanthanide oxide layer has a greater reducing effect on the transmittance of FTO samples than that for barrier glass-coated samples. Overall, the transmittance of the hydrophobic samples **A400**, **A450**, and **A500** were ∼50–80% in the visible region, with samples **A400** and **A450** achieving the highest transmittance (between 75 and 80%) and closest to that of bare FTO, used for photovoltaics. Consequently, **A400** and **A450** attained slightly higher reflectance measurements than FTO of 15–25% in the visible light region. These samples do therefore show potential for application in solar modules, provided that an improvement to the thickness of the coating, obstructing natural light, is made.

## 4 Conclusions

This work details the successful synthesis of cerium oxide hydrophobic coatings via AACVD of a presynthesized precursor [Ce­(thd)_4_]. AACVD was performed at temperatures of 400, 450, and 500 °C under a constant flow of dinitrogen onto FTO-coated and barrier glass substrates. The films deposited on FTO glass substrates demonstrated intrinsic hydrophobicity, with the highest deposition temperature resulting in the synthesis of the most hydrophobic film at 500 °C. This sample showed the presence of fully oxidized Ce­(IV) on the surface with a compact, granular, rough surface morphology. A comparison between the deposition of ceria using the same parameters except with barrier glass as the deposition substrate was also made. Here, it was found that an increase in the water contact angle and deposition temperature was still apparent. However, the barrier glass was overall insufficient in providing the highly textured material required for the synthesis of hydrophobic films like that displayed by the lotus leaf. The hydrophobic samples were analyzed by various surface characterization techniques such as XRD, XPS, SEM, and UV–vis. It was found that exposure to the atmosphere increased the water contact angle. Overall, the production of hydrophobic, self-cleaning glass for the cover glass of solar modules has been achieved via the deposition of the rare Earth oxide cerium oxide.

## Supplementary Material



## Notes

Thermogravimetric analysis of [Ce­(thd)_4_]; water contact angle data after air exposure (**A400, A450, A500**); contact angle hysteresis measurements; and optical absorbance and reflectance spectra (PDF)
