# Supplementary material for: Tetrakis(2,2,6,6-tetramethyl-3,5-heptanedionate) Cerium for the Deposition of Hydrophobic Coatings
Source: Langmuir. 2025 Jun 11;41(24):15562–72. doi: 10.1021/acs.langmuir.5c01723 (PMC12199470; doi:10.1021/acs.langmuir.5c01723)
Supplement: Supplementary file 1 [file la5c01723_si_001.pdf]

Supporting Information

Tetrakis(2,2,6,6-tetramethyl-3,5-heptanedionate) cerium for the deposition of hydrophobic coatings

Authors:

Jayna K. Patel, Iqra Ramzan, Cesar III De Leon Reyes, Ivan P. Parkin and Claire J. Carmalt*

Affiliations:

Materials Chemistry Centre, Department of Chemistry, University College London, WC1H 0AJ, United Kingdom

Corresponding author. Email: c.j.carmalt@ucl.ac.uk

Figure S1. Thermogravimetric analysis (TGA) of Tetrakis(2,2,6,6-tetramethyl-3,5-heptanedionate) cerium [Ce(thd)$_4$] used for the deposition of cerium oxide thin films via AACVD.

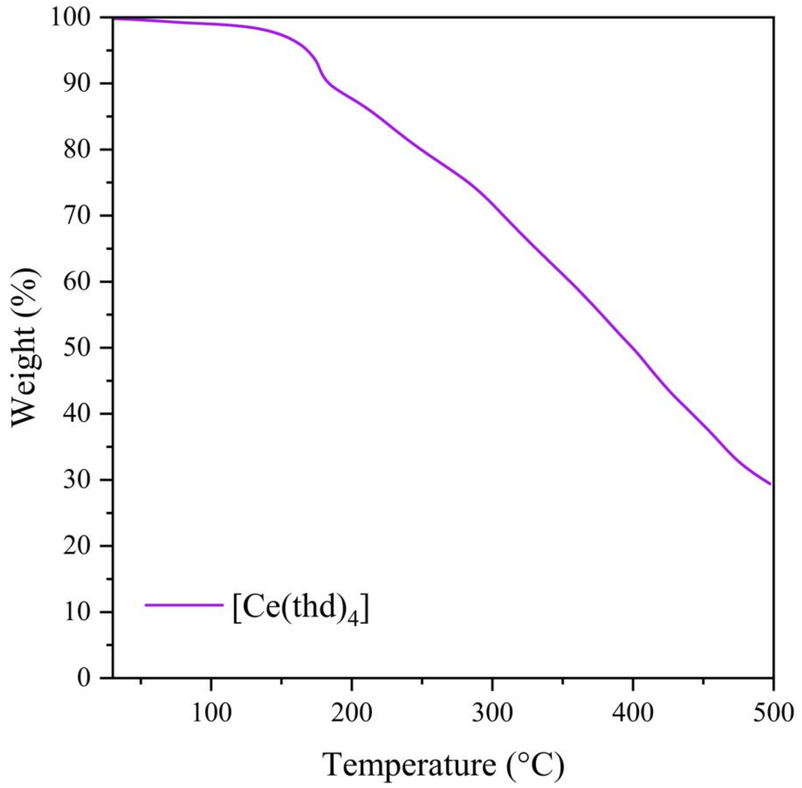

Figure S2. WCA measurements of hydrophobic cerium oxide coatings deposited onto FTO glass (**A400**-**A500**), before and after 30-day exposure of the films to the atmosphere.

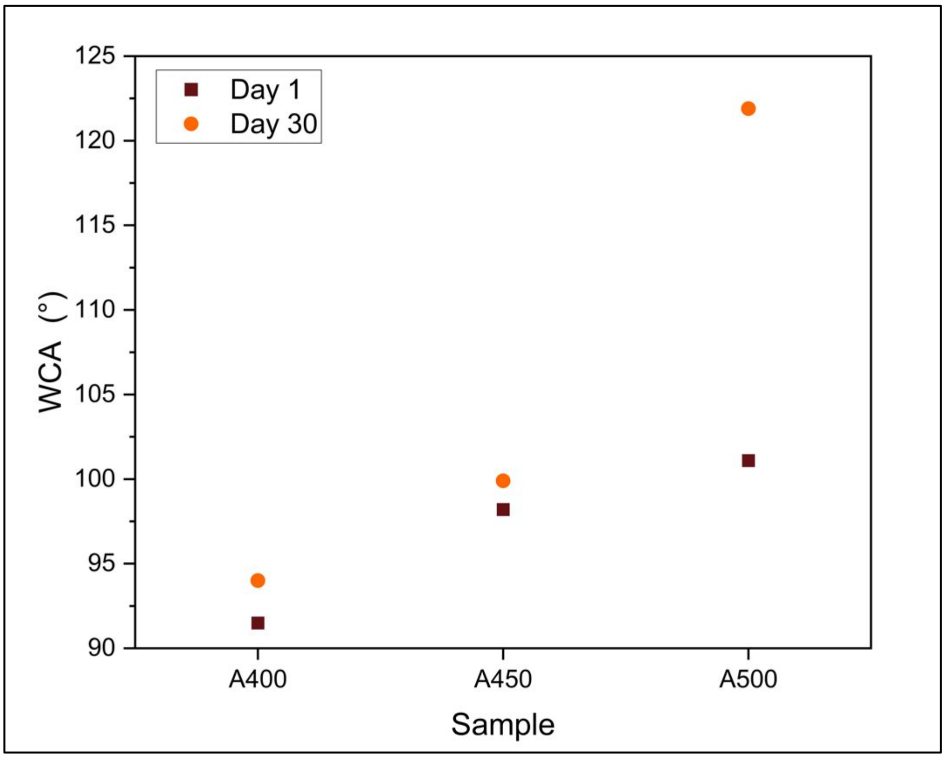

Figure S3. Optical reflectance measurements for samples **A400**, **A450 and A500** against that of bare FTO, with the visible light range (380-700 nm) highlighted.

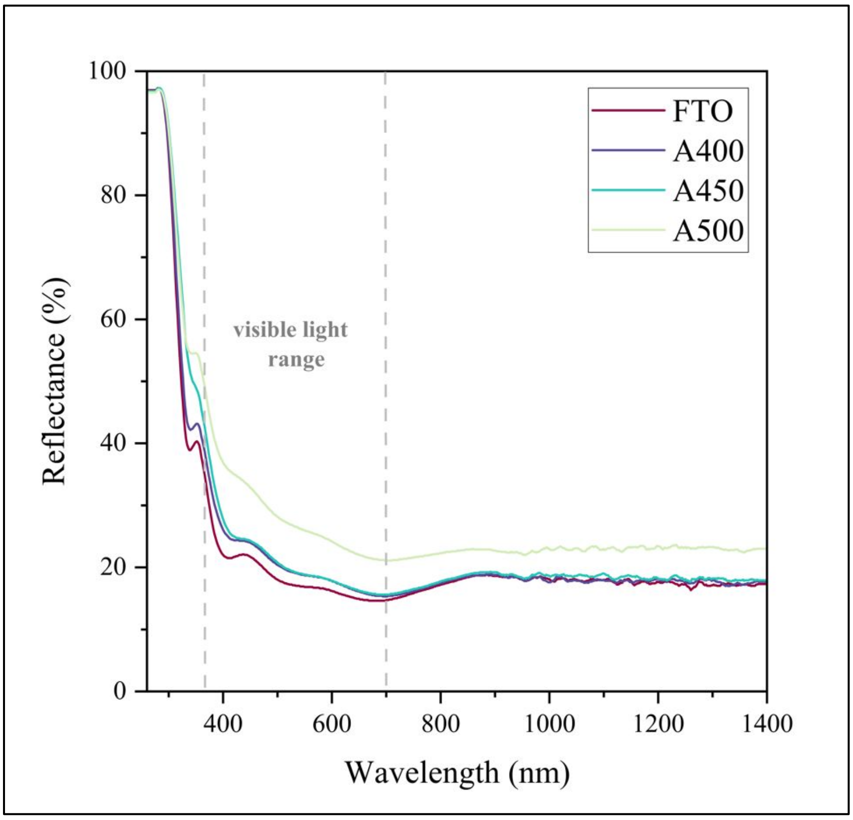

Figure S4. Optical reflectance measurements for samples **B400, B450 and B500** against that of bare barrier glass, with the visible light range (380-700 nm) highlighted.

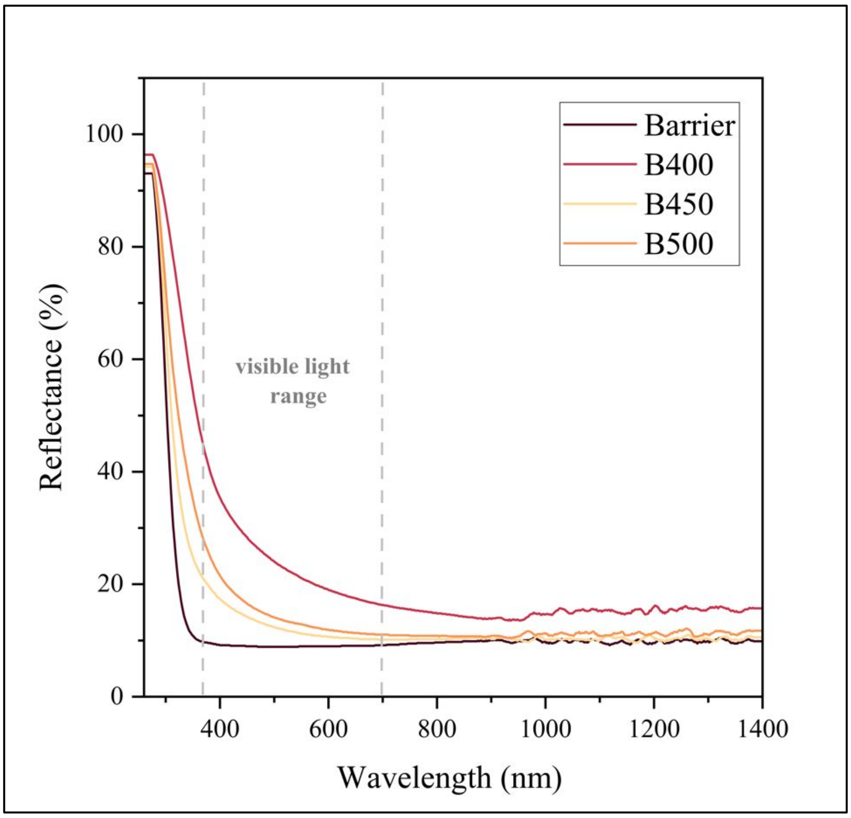

Figure S5. Optical absorbance measurements for samples **A400, A450 and A500** against that of bare FTO, with the visible light range (380-700 nm) highlighted.

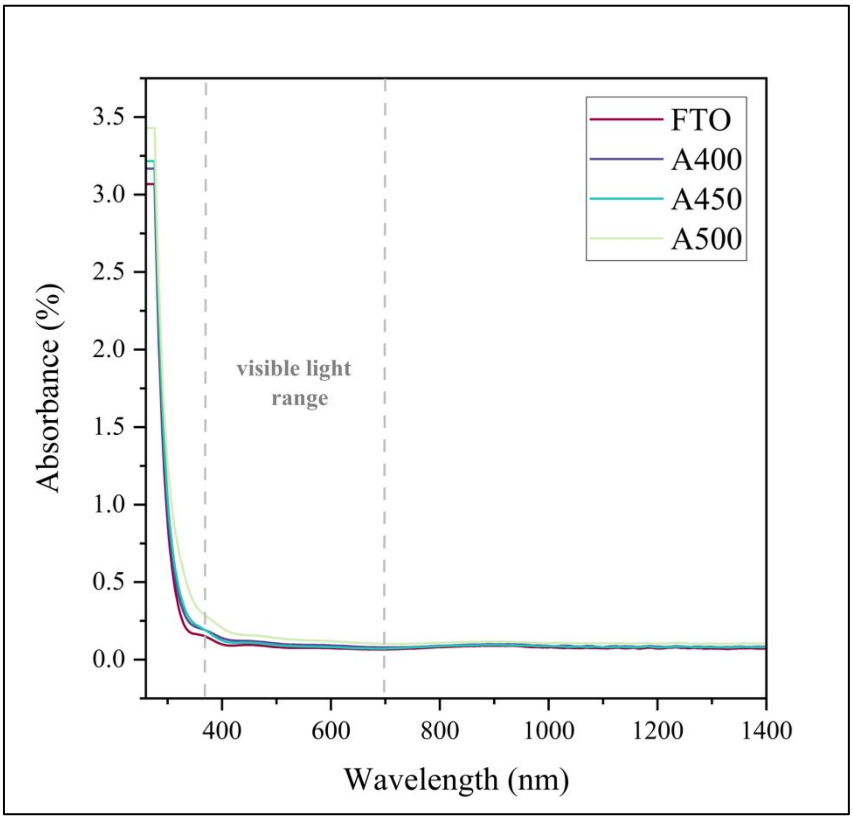

Figure S6. Optical absorbance measurements for samples **B400, B450 and B500** against that of bare FTO, with the visible light range (380-700 nm) highlighted.

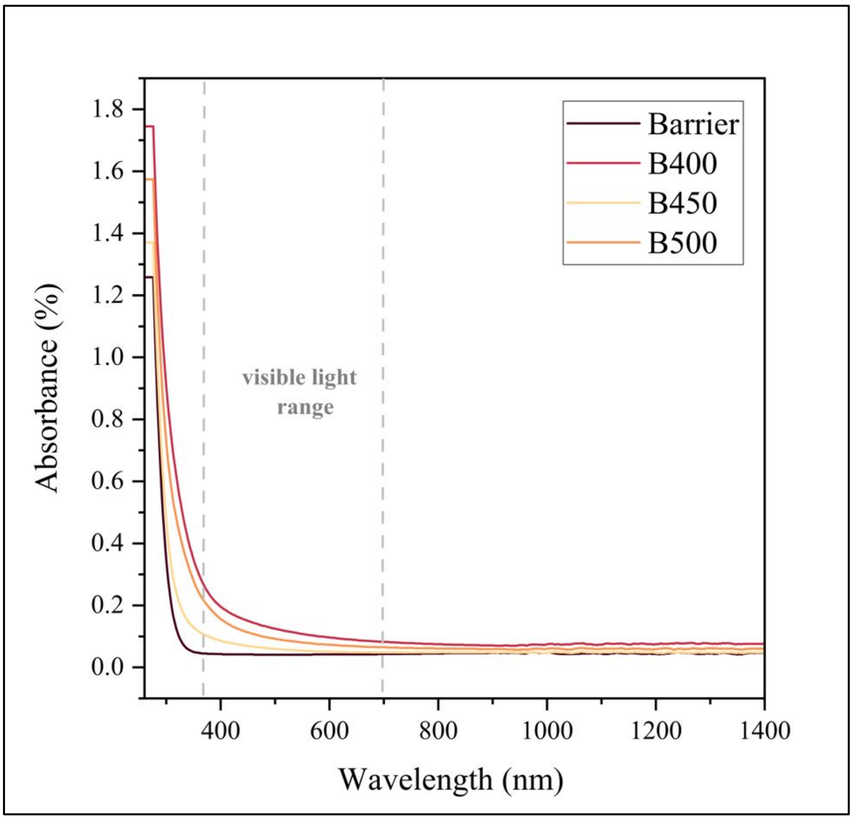

Table S1. Table detailing the advancing and receding water contact angle (CA) and calculated contact angle hysteresis (CAH) for cerium oxide films **A400, A450 and A500.**

Sample	Advancing CA (°)	Receding CA (°)	CAH (°)
A400	93.9	56	37.0
A450	105.4	33.7	71.7
A500	121.0	45.4	75.6
